# Radiation dose-fractionation effects in spinal cord: comparison of animal and human data

**DOI:** 10.1007/s13566-015-0212-9

**Published:** 2015-08-14

**Authors:** Jian-Yue Jin, Yimei Huang, Stephen L. Brown, Benjamin Movsas, Joseph Kaminski, Indrin J. Chetty, Samuel Ryu, Feng-Ming (Spring) Kong

**Affiliations:** Department of Radiation Oncology, Georgia Regents University, Augusta, GA 30912 USA; Department of Radiation Oncology, Henry Ford Hospital, Detroit, MI 48202 USA; Department of Radiation Oncology, Stony Brook University, Stony Brook, NY 11794 USA

**Keywords:** Cord dose tolerance, α/β ratio, Myelopathy, Dose fractionation effect

## Abstract

**Purpose:**

Recognizing spinal cord dose limits in various fractionations is essential to ensure adequate dose for tumor control while minimizing the chance of radiation-induced myelopathy (RIM). This study aimed to determine the α/β ratio of the spinal cord and the cord dose limit in terms of BED50, the biological equivalent dose (BED) that induces 50 % chance of RIM, by fitting data collected from published animal and patient studies.

**Methods:**

RIM data from five rat studies; three large animal studies on monkeys, dogs, and pigs; and 18 patient studies were included for the investigation. The α/β ratios were derived, respectively, for rat (group A), large animal (group B), patient (group C), and combined data (group D).

**Results:**

The α/β ratio (and its 95 % confidental interval) was 4.1 (3.2, 5.0) or 3.6 (2.6, 4.6) Gy for group A, depending on fitting algorithms. It was 3.9 (3.0, 4.8), 3.7 (2.2, 8.2) and 3.9 (3.0, 4.9) for groups B, C, and D, respectively. BED50 was 111 Gy for the combined data. It corresponds to a D50 of 73.4 Gy in 2 Gy/FX, or 19.0 Gy in single fraction. BED5, which is the BED to induce 5 % of RIM, was calculated to be 83.9 Gy. It corresponds to D5 of 55.4 Gy in 2 Gy/FX, or 16.2 Gy in single fraction.

**Conclusion:**

The study showed that all four groups had similar α/β ratios close to 3.9 Gy, suggesting that the spinal cord has a similar fractionation effect for different species, including human beings.

## Introduction

Radiation-induced myelopathy (RIM) is one of the most severe complications for radiation treatment of cancers in the neck, thoracic, and abdominal regions. The human spinal cord was estimated to have a probability of RIM of less than 5 % in 5 years following a dose of 47–50 Gy delivered in a standard 1.8–2Gy/fraction schedule [1, 2]. The spine is also one of the most frequent sites for metastases. The metastatic tumor often invades into the spinal cord, compresses the cord, and causes an emergency, which requires radiation treatment and surgical intervention. A conventional radiation regimen of 30 Gy in 10 fractions is often used to manage patients with cord compression, preceded or followed by a decompression surgery. Recently, a radiosurgical decompression approach has been proposed to manage these patients [3]. In addition, linear accelerator-based stereotactic radiosurgery (SRS) and stereotactic body radiotherapy (SBRT) have been frequently used to treat spine metastasis without cord compression [3–5]. A RIM model that reflects the fractionation effect of the spinal cord is needed to optimize the radiation dose and fractionation scheme.

The fractionation effect, represented by the α/β ratio in the linear quadratic model, has been well studied for the spinal cord on rats [6–10]. These prospective studies showed that the α/β ratio of the rat spinal cord ranged from 1.8∼4.6 Gy, depending on the site (cervical or lumbar spine) and the study [11]. However, applying rat models to humans is intrinsically complicated because their anatomy and lifespan are quite different from humans. The rat data may also have relatively large experimental uncertainties due to very narrow radiation fields used to spare the normal structures. The narrow radiation field usually has a non-flat dose profile across the field. Thus, even a normal experimental setup uncertainty may induce a relatively large dosimetric uncertainty.

Studies have been carried out on larger animals, such as dogs, monkeys, and pigs, for the RIM in several different fractionation schemes 12. However, large animal studies are expensive. The data from each of these experiments were not sufficient to derive the α/β ratio. Many retrospective studies have reported RIM incidences in humans at various cord doses and fractionations. An α/β ratio of 0.87 Gy, with a 95 % confidence interval (CI) of (0.54, 1.19) Gy was derived from these limited data for the human cervical spine [12]. However, human data are usually highly limited in power, because of the rare occurrence and nature of retrospective documentation. These data are often anecdotal, have limited number of data points, small range of doses, inconsistent endpoints, and sometimes, inaccurate cord dose calculations. For these reasons, the α/β ratio derived from these data may be unreliable.

The aims of this study were to compare the α/β ratios of the spinal cord between patients and animals by re-analyzing existing data in the literature, and to develop a RIM model from the combined data. Because the anatomy and life span between species is different, the animal data were further separated into small animal (rat) and large animal groups for comparison. Several unique approaches were used in the re-analysis: (1) normalization and non-linear regression were used to pool all rat data together to increase the statistic power and reduce the effect of large dosimetric uncertainties; (2) data of large animal were grouped together to derive an α/β ratio for this group; and (3) Monte Carlo simulation was used to correct a potential dosimetric error for one patient study, which provided the key data for deriving the α/β ratio on patients.

## Material and methods

### Data collection and correction

All available animal and patient studies were collected from literature searches using key words of “radiation induced myelopathy,”, “radiation, myelopathy,” “radiation, spinal cord complication,” or “radiation, spinal cord injury.” The data were separated into four groups: (A) small animals, (B) large animals, (C) patients, and (D) patients and large animals combined. To avoid the confounding effect of dose rate and volume effects, studies (or data points) with low dose rate (<0.1 Gy/min) or small volume (<2 vertebral sections) were excluded. Because of the concern of large dosimetric uncertainties in small animal studies (rats and mice), small animal data were not included in the combined patient and animal group, and only those rat studies specifically designed for studying the fractionation effect were included in the small animal group. A total of 5 rat [6–10], 3 large animal [13–15], and 18 patient studies [16–31] were finally included in this investigation.

The data of five rat studies [6–10] in group A were shown in Table 1. They had six data sets, with the corresponding data points expressed as (D50, *n*), where D50 is the total dose with 50 % of RIM incidence, and *n* is the fraction number. The data sets were further separated into the cervical (C) and thoracicolumber (T/L) spine subgroups. We noted that even within the same subgroup, there were large variations between different data sets. Two sources of experimental errors might contribute to these variations: (1) dose calibration uncertainty in the small animal experiments and (2) uncorrected quality factor between kilovolt and megavolt photons. The quality factor could be up to 1.12 for 50 kV photons [32].

**Table 1:** Data points of six data sets in five studies on rats specifically designed for the fractionation effect

Study	Radiation fields	Follow-up time (days)	Number of data points	Data points (D50 [Gy], fraction [*n*])
Karger (C) [6]	PA	300	4	(24.5, 1); (34.3, 2); (57, 6); (88.6, 18)
Ang (C2-T2) [7]	PA	210	4	(22.4, 1); (30.4, 2); (43.4, 4); (63, 10)
Van der Kogel (C) [8]	Lateral	∼365	5	(19, 1); (27, 2); (37.8, 5); (55, 10); (80, 30)
White (L) [9]	Lateral	365	6	(24, 1); (33, 2); (46, 4); (59, 8); (68, 15); (92, 30)
Masuda (T) [10]	Lateral	455	3	(25.5, 1); (34.5, 2); (46.6, 4)
Van der Kogel (L) [8]	Lateral	∼365	7	(19.5, 1); (27, 2); (32.1, 3); (36.8, 5); (47.7, 10); (60.4, 15); (67.1, 20)

*C* cervical, *T* thoracic, *L* lumber

Normalization was performed for these data to reduce the variations among different studies so they could be analyzed together. This was based on the assumption that all the rats had similar radiation response after a single fraction of irradiation, and the variation among different studies was due to experimental uncertainties (dose calibration and the uncorrected quality factor). The average D50 value of all data sets at single fraction was first calculated (it was 22.5 Gy). The normalization was accomplished by multiplying a factor for each data set so that the D50 values at single fraction were all equal to the average value.

Group B included three animal studies on dogs, monkeys, and pigs [13–15]. Several data points in the dog and monkey studies were not included in the analysis because the irradiated cord length could have been less than two vertebral sections. As shown in Table 2, the data were presented as the percentage of RIM (PRIM) incidence, and the irradiated dose per fraction (*d*) multiplying the number of fractions (*n*), or *d* × *n*.

**Table 2:** Data points from 3 studies on large animals and 17 studies on patients for the radiation-induced myelopathy model

Specie, location	Study	Data points (PRIM, *d* (Gy) × *n*)
Dog, T	Powers [14]	(3/12, 4 × 11); (6/12, 4 × 13); (17/17, 4 × 17)
		(2/12, 2 × 30); (1/6, 2 × 34); (3/6, 2 × 38); (5/6, 2 × 42)
Monkey, C1-T2	Schultheiss [15]	(3/15, 2.2 × 32); (3/6, 2.2 × 35); (7/8, 2.2 × 38); (6/16, 2.2 × 32)
Pig, C4-7	Medin [13]	(0/5, 16 × 1); (1/5, 18 × 1); (4/5, 20 × 1); (4/4, 22 × 1); (4/4, 24 × 1)
Human, C	Reinhold [34]	(0/2, 2.1 × 22); (1/5, 2.1 × 26); (2/4, 2.2 × 28); (5/9, 2.2 × 30); (0/2, 2.5 × 20); (2/4, 2.5 × 25); (4/6, 2.5 × 28); (4/9, 3 × 21); (2/2, 3.5 × 18)
Human, C (QUANTEC)	McCunniff [16]	(1/12, 2 × 30)
	Jeremic [17], McCunniff [16]	(0/24 + 0/19, 1.63 × 40) = (0/43, 1.63 × 40)
	Abbatucci [18]	(7/15, 3 × 18) → excluded from study
	Atkins [19]	(4/13, 9.5 × 2) → (4/13, 12 × 2)
	Marcus [20]	(0/211, 1.9 × 25)
		(0/22, 1.9 × 28)
		(2/19, 2 × 30)
Human, T (QUANTEC)	Hazra [21] Choi [22]	(1/16 + 0/75, 3 × 15) = (1/91, 3 × 15)
	Abramson [23] Fitzgerald [24] Madden [25] Guthrie [26]	(4/271 + 6/45 + 1/43 + 0/42, 4 × 10) = (11/401, 4 × 10)
	Dische [27]	(13/145, 5.7 × 6)
	Haltlevoll [28]	(8/157, 6 × 3 + 4 × 5)
		(9/230, 6 × 3 + 4 × 3 + 2 × 2)
	Eichhorn [29]	(8/142, 2.45 × 27) → (8/46, 2.45 × 27)
	Scruggs [30]	(2/248, 4 × 5 + 2.5 × 8)
	Macbeth [31]	(3/524, 9.2 × 2)
		(2/153, 3.1 × 13)

*PRIM* percentage of radiation-induced myelopathy = number of RIM/total number; *d* dose/fraction, *n* number of fractions, *C* cervical, *T* thoracic

The patient data in group C had a total of 25 data points (RIM data at 25 different fractionation regimens). The data were also presented as (PRIM, *d* × *n*) in Table 2. Among them, 16 data points were from 16 patient studies [16–31] previously compiled and analyzed for the α/β ratio [12, 35]. These data were also reviewed in a Quantitative Analysis of Normal Tissue Effects in the Clinic (QUANTEC) paper [2]. Several of these studies had more than one data points, and others had the same dose-fractionation regimen, which were combined into a single data point. For the 16 data points, 7 were on C-spine, and 9 on thoracic (T)-spine. The other 9 data points were new data from a set of patient data collected in four hospitals in Netherlands [34]. That study reported that 19 of 43 patients developed myelopathy in the T spinal cord with the radiation dose ranging from 43 to 74 Gy, and fraction size ranging from 1.9 to 3.6 Gy/fraction. The 43 patients were divided into nine groups according to the doses and fractionations. Patient data from spinal SRS or SBRT [2] were not included in this study due to the patient setup-related dosimetric uncertainty and the partial cord volume irradiation.

**Fig. 1:**
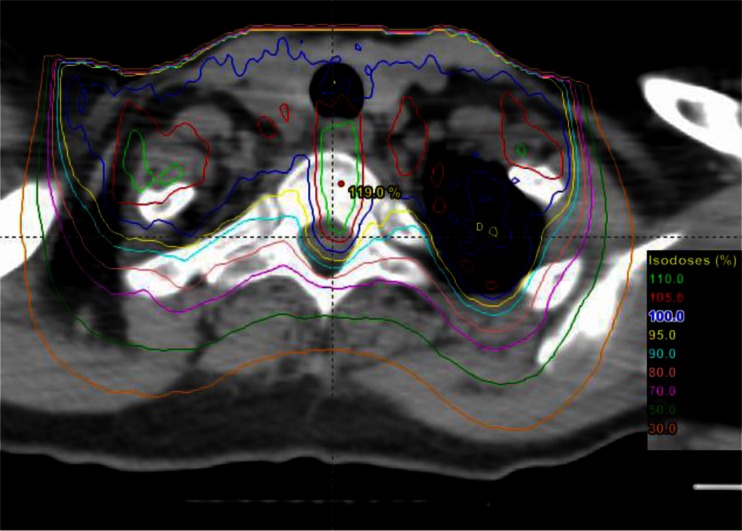
Monte Carlo calculated dose distribution of an AP 20 MeV electron beam to a female patient

All the patient data were thoroughly evaluated with the original sources. Of note, three data points were modified as a result. Data point 1 was from a report of myelopathy after radiotherapy of breast cancers [19]. A 22.5 MeV anterior-posterior electron beam was given to the chest wall and supraclavicular area. The prescription dose was 25 Gy in 2 fractions. The cord dose was recorded as 19 Gy in 2 fractions. However, a Monte Carlo calculation suggested that the cord dose could be much higher. Figure 1 shows the dose distribution with Monte Carlo calculation of a 20-MeV electron beam in AP direction delivered to the same region for a female patient. We noted that the maximum cord dose was as high as the prescription dose. Therefore, the cord dose for data point 1 was estimated to be 24 Gy in 2 fractions.

Data point 2 was from Eichhorn et al. [29]. The incidence of RIM was first listed as 8/46 [35], but might be subsequently misquoted as 8/142 in later papers [2, 12]. The correct value, which is 8/46, was used in our study. Data point 3 was from a French study [18]. Incidence of RIM was first listed as 8/20 [35] and then 7/15 in later papers [2, 12] for a cord dose of 3-Gy × 18. However, there were 94 patients who received a 3 Gy × 18 prescription dose in the control group [18]. Because the cord dose of these 94 patients was not clearly provided, the incidence of RIM for this data point was difficult to verify and the data point was excluded from the final analysis.

### Data analysis

For group A, a conventional linear regression method [11] was first used to derive the α/β ratio as well as the biological equivalent dose (BED) at 50 % of RIM probability (BED50). The linear equation was derived directly from a BED equation,

1 $$ \mathrm{BED}50=\mathrm{D}50\cdot \left[1+\frac{\mathrm{D}50}{n\cdot \left(\raisebox{1ex}{$a$}\!\left/ \!\raisebox{-1ex}{$\beta $}\right.\right)}\right] $$

Dividing both sides of Eq. (1) to a term of D50•BED50, we have,

2 $$ \frac{1}{\mathrm{D}50}=\frac{1}{\mathrm{BED}50}+\frac{1}{\mathrm{BED}50\cdot \left(\raisebox{1ex}{$a$}\!\left/ \!\raisebox{-1ex}{$\beta $}\right.\right)}\cdot \frac{\mathrm{D}50}{n} $$

Equation (2) is a linear function of (1/D50, D50/*n*). The data set of (D50, *n*) was converted into (1/D50, D50/*n*) for linear regression analysis. The values of BED50 and α/β were derived by best fitting the data into the linear equation. Such analysis was performed for each data set for the original data, and the combined data set for each subgroup after normalization.

The linear regression approach had to convert the data point (D50, *n*) into (1/D50, D50/*n*), and thus propagate the large uncertainty in D50 into both variables. To reduce the effect of such data conversion, a non-linear regression approach was also developed to derive the values of BED50 and α/β. The data set of (D50, *n*) was directly fitted into a non-linear equation,

3 $$ \mathrm{D}50=\left\{{\left[{\left(\alpha /\beta \right)}^2\cdot {n}^2-4n\cdot \alpha /\beta \right]}^{1/2}-n\cdot \mathrm{BED}50\cdot \alpha /\beta \right\}/2 $$

which is the direct solution of Eq. (1).

For groups B, C, and D, the following logistical RIM model was used to fit the data:

4 $$ \mathrm{PRIM}\left(d,n\right)=1/\left[1+{\left(\mathrm{BED}50/\mathrm{BED}\right)}^k\right]=1/\left[1+{\left(\frac{\mathrm{BED}50}{d\cdot n\cdot \left(1+d/\alpha /\beta \right)}\right)}^k\right], $$

It should be noted that for several patient data points, multiple fractionation regimens were used in a treatment course. The sum of BED in each fractionation regimen was used for Eq. (4) in such situations. The sum of BED was expressed as

5 $$ \mathrm{BED}={\displaystyle \sum_{i=1}^m{d}_i\cdot {n}_i\cdot \left(1+{d}_i/\alpha /\beta \right)} $$

where *d*_*i*_ and *n*_*i*_ are the dose and fraction number of the *i*th regimen, and *m* is total number of regimens. Three parameters, α/β, BED50, *k*, and their corresponding 95 % CI were thus determined from the fitting program.

## Results

### Fractionation effect from the rat studies

**Fig. 2:**
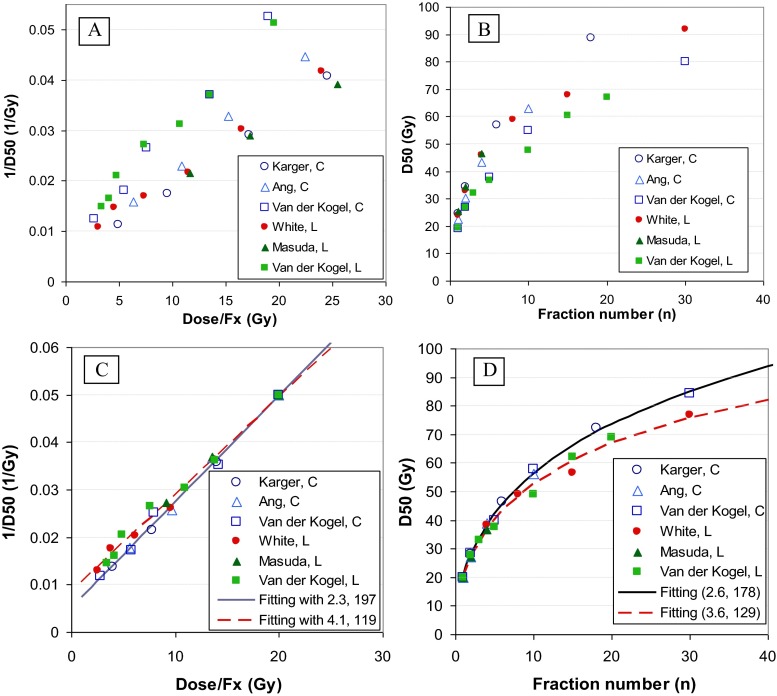
Re-analysis of the α/β ratio for the six data sets from five rat studies specifically designed for studying fractionation effect using linear and non-linear regression. **a** Original data plotted (1/D50, D50/*n*) for linear regression. **b** Original data plotted (D50, *n*) for non-linear regression. **c** Normalized data plotted (1/D50, D50/*n*) and linear regression of the combined data for C- and T/L-spine subgroups. **d** Normalized data plotted (D50, *n*) and non-linear regression of combined data sets for the two subgroups

Figure 2a, b show the original six data sets of the rat studies (group A) plotted as (1/D50 vs D50/*n*) for linear regression, and (D50 vs *n*) for non-linear regression, respectively. Figure 2c, d show the same data after normalization, as well as their linear and non-linear regressions, respectively. The large inter-study variations in Fig. 2a, b were dramatically reduced after normalization. The data were clearly separated into C-spine and T/L-spine subgroups, and the inter-study variation within each subgroup was comparable to the intra-study variation.

Table 3 shows the values of α/β ratio and BED50 after regression analyses. Despite large inter-study variation in the original data, the α/β ratios were relatively consistent within each subgroup. The average α/β ratios were 2.4 and 4.8 Gy for the C-spine and T/L-spine subgroups, respectively, before normalization. They were slightly reduced to 2.2 and 4.1 Gy after normalization. The overall α/β ratios and their 95 % CI derived from the combined data sets were 2.3 (1.5, 3.0) Gy and 4.1 (3.2, 5.0) Gy, respectively, for the C- and T/L-spine subgroups, using the linear regression. They became 2.6 (2.1, 3.2) Gy and 3.6 (2.6, 4.6) Gy, respectively, using the non-linear regression. In summary, the α/β ratios were slightly changed by the normalization and the different regression approaches, and consequently, the difference in α/β ratios between the two subgroups (C- and T/L-spines) was reduced, especially using the non-linear regression. As to be discussed, the smaller α/β ratio in the C-spine subgroup could be due to shorter follow-up time. Thus, the values of the T/L-spine subgroup would be used for comparison to the large animal and patient data.

**Table 3:** Values of α/β ratio and BED50 from six data sets in five studies with and without normalization. The data were divided into cervical (C)-spine and thoracicolumber (T/L) spine groups

Subgroup	Study	Regression	Original		After normalization	
			α/β (Gy)	BED50 (Gy)	α/β (Gy)	BED50 (Gy)
C-Spine	Karger [6]	Linear	2.3	286	1.9	234
	Ang [7]	Linear	2.3	238	2.1	211
	Van der Kogel [8]	Linear	2.5	167	2.6	174
	Average	Linear	*2.4*	*230*	*2.2*	*207*
	Overall	Linear	*NA*	*NA*	*2.3* (*1.5*, *3.0*)	*198* (*142*, *235*)
	Overall	Non-linear	*NA*	*NA*	*2.6* (*2.1*, *3.2*)	*178* (*153*, *203*)
T/L-Spine	White [9]	Linear	4.8	149	3.9	124
	Masuda [10]	Linear	5.2	149	4.1	117
	Van der Kogel [8]	Linear	4.3	111	4.3	114
	Average	Linear	*4.8*	*136*	*4.1*	*118*
	Overall	Linear	*NA*	*NA*	*4.1* (*3.2*, *5.0*)	*119* (*100*, *138*)
	Overall	Non-linear	*NA*	*NA*	*3.6* (*2.6*, *4.6*)	*129* (*109*, *150*)

### Fractionation effect from large animal and human data

**Fig. 3:**
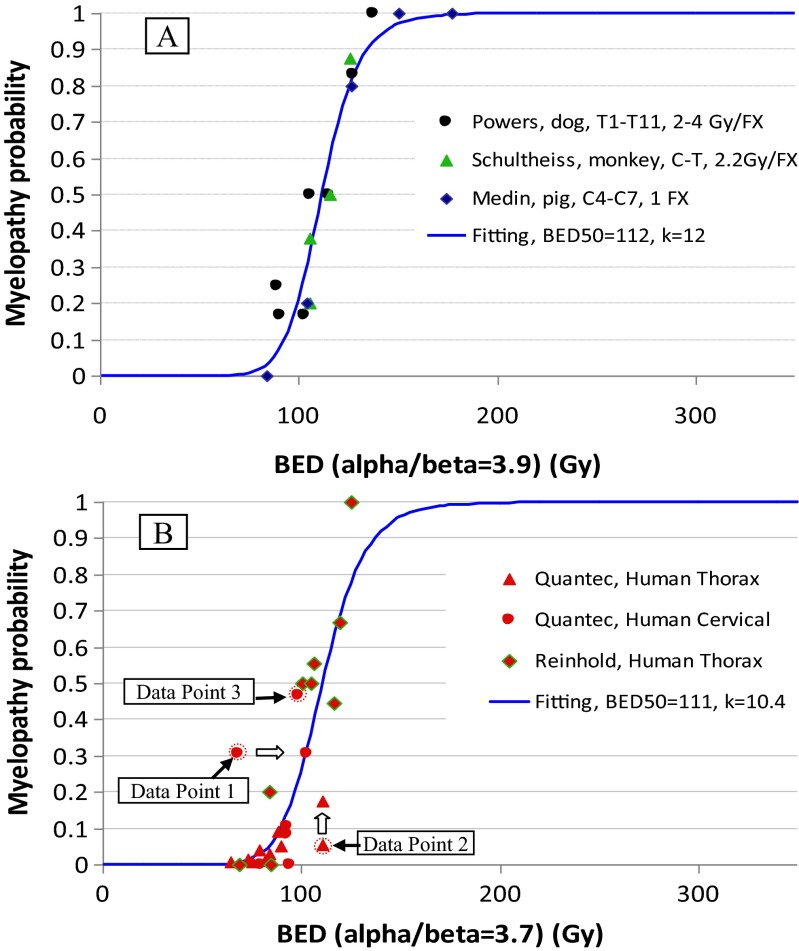
Fitting the data with a logistic radiation-induced myelopathy (RIM) model. The data are plotted as percentage of RIM versus BED. BED was calculated from dose/fraction and number of fractions using the best fitting α/β ratio. **a** Large animal data. **b** Patient data. Data points 1, 2, and 3 before correction were also plotted for comparison

Figure 3a , b show the plots of the large animal (group B) and patient data (group C) with the best fitting of the RIM model. The original data points 1, 2, and 3 before correction were also shown in Fig. 3b for comparison. The best fitting parameters and their 95 % CI were α/β = 3.9 (3.0, 4.8) Gy, BED50 = 112 (101, 128) Gy, and *k* = 12 (7.0, 21) for group B; and α/β = 3.7 (2.2, 8.2) Gy, BED50 = 111 (86, 148) Gy, and *k* = 10.4 (5.8, 19.2) for group C. These data were also listed in Table 4 for comparison between the four groups. The results suggest that the rats, large animals, and patients have very similar α/β ratios. We also noted that the patient data had a large and unbalanced 95 % CI for all parameters. This is understandable because the data set was unbalanced for the fractionation size. Only 2 data points had a large dose/fraction.

**Table. 4:** Comparison of the fitting parameters for groups A, B, C and D

Groups	Regression	α/β (Gy)	BED50 (Gy)	*k*
A (rats)	Linear regression	4.1 (3.2, 5.0)	119 (100, 138)	NA
	Non-linear regression	3.6 (2.6, 4.6)	129 (109, 150)	NA
B (large animals)	Non-linear regression	3.9 (3.0, 4.8)	112 (101, 128)	12 (7.0, 21.0)
C (patients)	Non-linear regression	3.7 (2.2, 8.2)	111 (86. 148)	10.4 (5.8, 19.2)
D (combined patients and large animals)	Non-linear regression	3.9 (3.0, 4.9)	111 (101, 120)	10.5 (6.8, 14.2)

### RIM model with fractionation effect from the combined data

**Fig. 4:**
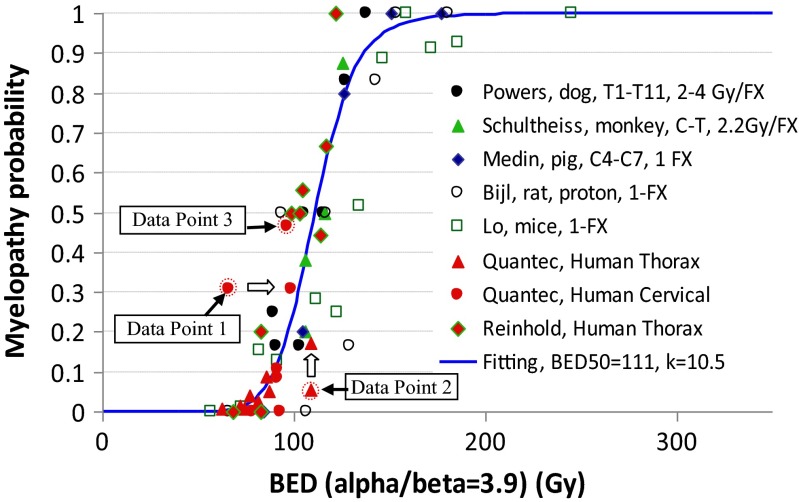
Fitting the combined (group D) data with a logistic radiation-induced myelopathy (RIM) model. In addition to the 3 patient data points before correction, data from mice and rat studies were also plotted for comparison

Figure 4 shows the plot of the combined patient and large animal data (group D) with the best fitting of the RIM model. In addition to the 3 original patient data points before correction, animal data from two studies on mice [36] and rats [37] were also presented for comparison. The best fitting parameters and their 95 % CI were α/β = 3.9 (3.0, 4.9) Gy, BED50 = 111 (101, 120) Gy, and *k* = 10.5 (6.8, 14.2). We noted that combining the large animal and patient data did not change the best fitting parameters much, while the 95 % CIs for BED50 and *k* were remarkably narrowed, which is essential for the applicability of the RIM model. We also noted that the rat and mice data tended to agree with the patient data. However, although the mice or rat data set was from a single experiment, the data were more spread out than the patient and large animal data, possibly due to larger dosimetric uncertainties in small animals.

## Discussion

With the corrections for possible dosimetric and misquotation errors in patient data, and the normalization of the rat data, we have determined that the α/β ratios of the spinal cord were 3.6–4.1, 3.9, and 3.7 Gy for small animals (rat), large animals, and patients, respectively. We have also determined the three parameters (including their 95 % CI) for a logistic RIM model from the combined data. They were α/β = 3.9 (3.0, 4.9) Gy, BED50 = 111 (101, 120) Gy, and *k* = 10.5 (6.8, 14.2).

The α/β ratios are similar between different species, including human beings. These α/β ratios of 3.6–4.1 Gy, however, are greater than the previously reported 0.87 Gy [12]. That study had only 7 data points from five patient studies, including 3 data points with a same percentage of RIM of 0, and 2 data points in dispute (points 1 and 2 in the current study). The statistical power was limited when the data set was fitted to a non-linear equation with three parameters. The data point 1, which was from the study of breast treatment with 25 Gy in 2 fractions [19], was the most critical point to determine the α/β ratio. The change of α/β ratio from 0.87 Gy of that study to 3.7 Gy of the current study is likely due to the adjustment of the cord dose from 9.5 to 12 Gyx2. The cord dose of 12 Gyx2 was estimated based on Monte Carlo simulation in the current study, which is more reliable. The lower bound of the 95 % CI of the α/β ratio for the patient group was 2.2 Gy, which is similar to the 2.0 Gy value commonly used clinically for isoeffect calculations.

We noted that the α/β ratio was larger, and BED50 smaller, for the C-spine than the T/L-spine subgroups of the rat studies (Table 3). However, the C-spine subgroup had a shorter follow-up time. The latency of radiation myelopathy was reported to have a bimodal distribution peaking at approximately 9 and 26 months in humans [33]. In rats, the two peaks correspond to two distinct pathologies, with the first peak reflecting white matter injury occurring less than 8 months after radiation exposure, and the second peak reflecting vascular injury occurring between 8 and 18 months after radiation exposure [33]. The latent period depends on the radiation dose and fraction size [9, 36]. High-dose and single fraction tend to result in shorter latent periods [9, 36]. Therefore, longer follow-up time would record more events for lower dose at larger fraction numbers (*n*). As shown in Fig. 2d, this would shift the points of the C-spine subgroup at large *n* downward, and thus decrease BED50 and increase α/β ratios. Therefore, there is a possibility that the larger BED50 and smaller α/β ratio observed in the C-spine group was attributed to the shorter follow-up time rather than the anatomic location per se. The non-linear regression also reduced the difference between the two subgroups, suggesting that experimental uncertainty may also contribute to it. The patient data for C- and T-spine was similar, supporting the argument that there is no difference between the C- and T-spine.

The study showed a BED50 of 111 Gy for the combined and patient data. It corresponds to a D50 of 73.4 Gy in 2 Gy/FX, or 19.0 Gy in single fraction. BED5, which is the BED to induce 5 % of RIM, was calculated to be 83.9 Gy according to Eq. (4) with *k* = 10.5. It corresponds to D5 of 55.4 Gy in 2 Gy/FX, or 16.2 Gy in single fraction. However, applying these data to clinical SBRT is not straightforward due to two factors. The first factor is the dose volume effect. The cord dose is usually not uniform in spinal SRS or SBRT due to the use of intensity modulated techniques. The cord dose is represented by the dose volume histogram and has to be converted into an equivalent uniform dose. The second factor is the setup uncertainty in spinal SRS and SBRT. Even with image guidance, the combined patient setup uncertainty could have a 5 % probability of being greater than 3 mm due to patient position deformation, uncorrected rotation errors, and patient movement during treatment [38]. Such setup errors may shift the cord into a high-dose region due to the sharp dose falloff between the target and the cord. A RIM model incorporating the setup uncertainty, similar to the approach used in tumor control probability model by Jin et al. [39], may be required to improve the prediction of RIM for spinal SRS or SBRT patients.

## Conclusion

The study suggests that the spinal cord has a similar α/β ratio for patients, rats and other large animals, such as dogs, monkeys, and pigs. The α/β ratio is close to 3.9 Gy, with a 95 % CI of (3.0, 4.9) for the combined group, and (2.2, 8.2) for the patient only group.
